# Weight Gain and Risk of Impaired Fasting Glucose After Smoking Cessation

**DOI:** 10.2188/jea.JE20110010

**Published:** 2011-11-05

**Authors:** Mitsumasa Kamaura, Hitoshi Fujii, Shunsaku Mizushima, Osamu Tochikubo

**Affiliations:** 1 Department of Occupational Health, Kanagawa Health Service Association, Yokohama, Japan; 2 Department of Human Resource Development, National Institute of Public health, Saitama, Japan; 3 Department of Epidemiology and Public Health, Yokohama City University, Graduate School of Medicine, Yokohama, Japan

**Keywords:** smoking cessation, fasting, glucose tolerance, weight gain

## Abstract

**Background:**

Observation of early changes in fasting plasma glucose level induced by post-smoking cessation weight gain is useful in predicting the risks of diabetes mellitus (DM) and impaired fasting glucose (IFG). We investigated the effect of post-smoking cessation weight gain on early changes in the risk of a high fasting plasma glucose (IFG) level (≥100 mg/dL).

**Methods:**

In 946 subjects who underwent repeated health examinations after smoking cessation, changes in body mass index (BMI) and the odds ratio (OR) for IFG risk (adjusted for sex, age, BMI, fasting plasma glucose at year 1, and alcohol consumption) were calculated every year for 3 years after smoking cessation.

**Results:**

After smoking cessation, the rate of BMI increase significantly increased in quitters: 2.36% at year 2 (never smokers: 0.22%, current smokers: 0.39%) and 0.46% at year 3 (never smokers: 0.14%, current smokers: 0.32%). However, it decreased by 0.15% at year 4 (never smokers: 0.12%, current smokers: 0.26%). The ORs for quitters did not significantly increase at any time during the follow-up period. However, among quitters who had smoked at least 20 cigarettes per day, it was significantly higher (OR 1.51, 95% confidence interval 1.1–2.01 at year 1 and 1.71, 1.23–2.38 at year 2).

**Conclusions:**

The time course of the risk of IFG after smoking cessation was similar to that for the rate of BMI increase. In contrast to the findings of previous reports, the increase in IFG risk after smoking cessation was brief and disappeared in the absence of a significant increase in BMI.

## INTRODUCTION

Smoking cessation is associated with substantial weight gain,^1^^–^^3^ which can increase insulin resistance^4^ and the risk of diabetes mellitus (DM).^5^^–^^7^ However, some evidence indicates that cigarette smoking leads to insulin resistance^8^^–^^10^ and accelerates the development of DM.^11^^,^^12^ Although smoking cessation reduces the risk of DM, concerns about weight gain and the risk of impaired glucose tolerance (IGT) after smoking cessation are potential barriers to quitting smoking.^13^ Some studies have examined the effect of weight gain after smoking cessation on glucose tolerance^14^^–^^17^; however, few have examined the effect of such weight gain on impaired fasting glucose (IFG: fasting plasma glucose ≥100 mg/dL or ≥5.6 mmol/L).^18^^,^^19^ Observation of early changes in fasting plasma glucose (FPG) induced by post-smoking cessation weight gain is useful for predicting the risks of DM and IGT. In this study, we analyzed longitudinal data from workplace health examinations to examine the relationship between body mass index (BMI) and smoking status, the effect of the time interval since smoking cessation on BMI among quitters, and the influence of weight gain after smoking cessation on the risk of IFG.

## METHODS

A total of 30 285 workers were examined from April 1999 to March 2008 by the Kanagawa Health Service Association. The duration of follow-up was set at 4 years because it is believed that IFG occurs earlier than DM and that evaluation of annual changes during a shorter period after smoking cessation than that used in other studies (eg, 5 years to several decades) could be important with regard to prevention. This shorter follow-up period was also selected because most weight gain in quitters, as compared with nonsmokers and current smokers, occurs within 3 years of smoking cessation.^2^^,^^20^

**Figure 1.**
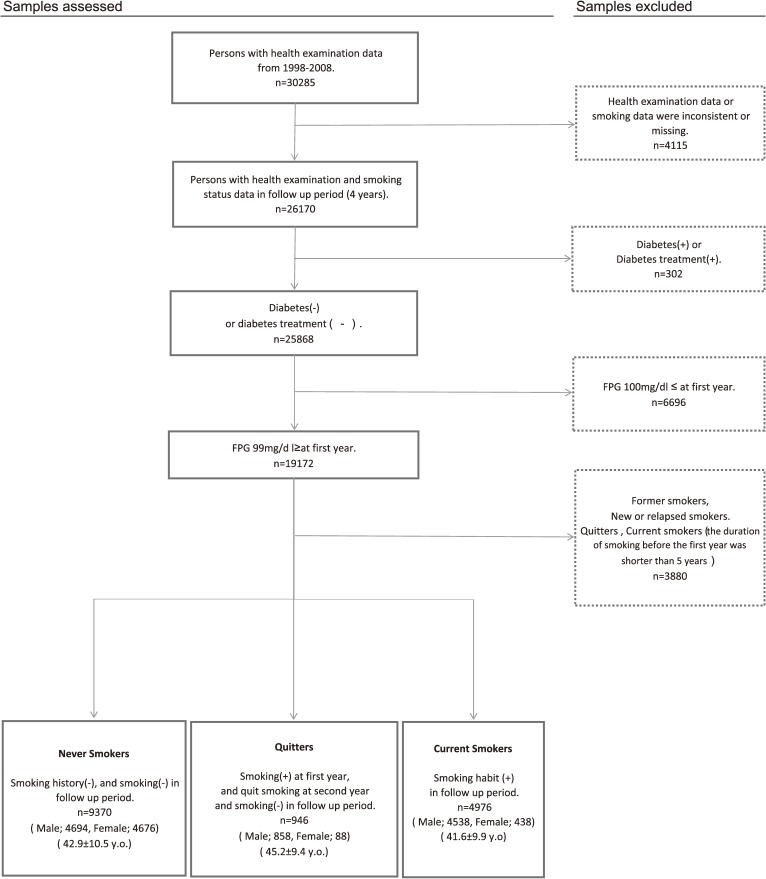
The process of sample selection (The study flow diagram)

Among subjects who underwent health examinations for 10 years (*n* = 30 285), data on health characteristics and smoking history over 4 or more consecutive years were available for 26 170. After excluding those with or being treated for diabetes, 25 868 subjects remained. To compare the risk of IFG for quitters with that of never smokers and smokers, subjects with an FPG concentration of 100 mg/dL or higher at year 1, former smokers, and new or relapsed smokers were excluded. Quitters and new smokers with a duration of smoking of less than 5 years before year 1 were excluded, and all remaining subjects (*n* = 15 292) were included in the survey (Figure 1).

### Smoking assessment

The questionnaire asked about smoking habits (never, past, or current smoker); current smokers were asked about the number of cigarettes smoked per day and the duration of smoking in years. Current smokers were subdivided into 2 groups based on the number of cigarettes smoked daily: 1 to 19 cigarettes/day or 20 or more cigarettes/day.

On the basis of the above criteria, smoking status was defined as follows: never smokers were subjects who had never smoked in the past. Quitters were subjects who had a 5-year or longer smoking habit at year 1 and had stopped smoking at year 2. Using their health examination data for 4 consecutive years, continuation of smoking cessation throughout the follow-up period was confirmed. Smokers were subjects with a 5-year or longer smoking habit at year 1 who were confirmed to have continued habitual smoking throughout the follow-up period.

This study was conducted in accordance with the Code of Ethics of the World Medical Association (Declaration of Helsinki, 1964, and Declaration of Tokyo, 1975, as revised in 1983), and the study protocol was approved by the Ethics Committee of the Kanagawa Health Service Association. Written informed consent was obtained from all subjects enrolled in this study.

### Other variables

Annual health examinations included medical history, physical examination, anthropometric measurements, biochemical measurements, and a questionnaire on health-related behaviors such as smoking and alcohol consumption. Medical history and history of prescription drug use were assessed by the examining physicians. Family history of diabetes was defined as a first-degree relative with diagnosed diabetes. BMI was used as a measure of overall obesity and was calculated as body weight/height^2^ (kg/m^2^).

With regard to health-related behaviors, the questions on alcohol intake included items on the type of alcoholic beverage, frequency of alcohol consumption per week, and usual amount consumed daily. Weekly alcohol intake was calculated and converted to daily alcohol consumption (grams of ethanol per day) by using standard Japanese tables.

Blood was collected from subjects after an overnight fast; samples were drawn from an antecubital vein. Blood tests were performed by using an automatic analyzer (JCA-BM9030; Nihon Denshi Co., Ltd. Japan) to determine FPG and hemoglobin A1c (HbA1c) levels. The reagents used were Pureauto S (Sekisui Medical Co., Ltd. Japan) for the measurement of FPG and RAPIDIA Auto HbA1c (Fujirebio Inc., Japan) for the measurement of HbA1c.

### Statistical analysis

First, the means for BMI, FPG, HbA1c, alcohol consumption, and rate of alcohol consumption during the follow-up period were calculated with respect to smoking status. Because weight gain and FPG are closely associated,^5^^–^^7^ the mean rate of BMI increase (the increase from the value in the previous year divided by the previous BMI value) was calculated. The significance of these values was analyzed by employing analysis of covariance, with sex and age as covariates in each year. The chi-square test was used to analyze rate of alcohol consumption by smoking status. The risk ratios for IFG among quitters and current smokers were calculated with never smokers as the reference group. In calculating risk ratios, the odds ratios (ORs) for each smoking status in years 2, 3, and 4, before and after adjustment for age, sex, BMI, FPG in year 1 and alcohol consumption per week, were calculated using logistic regression analysis. To calculate ORs, the risk of developing new IFG was evaluated, excluding those who developed IFG during the follow-up period. Also, as reference, the OR of IGT (FPG ≥110 mg/dL) was calculated using the same method. The OR of DM (FPG ≥126 mg/dL) was not calculated because it was extremely rare in quitters, as subjects at year 1 were limited to those with an FPG <100 mg/dL (Table 2). SPSS for Windows 19.0 was used for the statistical analysis.

**Table 1.** Characteristics of study subjects by examination year and smoking status

	No. of cigarettes/day	No.	1st year	2nd year	3rd year	4th year
Smoking status	Never Smoker	9370	(−)	(−)	(−)	(−)
	Quitter	946	(+)	(−)	(−)	(−)
	Current Smoker	4976	(+)	(+)	(+)	(+)

BMI (kg/m^2^)	Never Smoker		22.50 (3.0)	22.55 (3.0)^†^	22.60 (3.1)^†^	22.60 (3.1)^†^
	Quitter		22.85 (2.7)	23.37 (2.8)*	23.50 (2.8)*	23.56 (2.8)*
	<20/day		22.79 (2.8)	23.28 (2.8)*	23.31 (2.8)	23.28 (2.7)*
	≥20/day		22.97 (2.7)	23.53 (2.9)	23.73 (2.9)	23.86 (2.9)
	Current Smoker		22.97 (3.0)	23.02 (3.1)*	23.05 (3.0)*	23.10 (3.1)*
	<20/day		22.61 (3.0)	22.66 (3.0)	22.75 (3.0)	22.81 (3.0)
	≥20/day		23.09 (3.0)	23.16 (3.0)	23.19 (3.1)	23.24 (3.1)*

FPG (mg/dl)	Never Smoker		89.4 (6.0)^†††^	90.2 (6.9)^†††^	90.5 (7.4)^†††^	90.5 (7.4)^†††^
	Quitter		90.2 (6.1)***^†††^	92.8 (8.0)***^†††^	93.9 (9.2)***^†††^	93.5 (8.6)***^†††^
	<20/day		90.0 (6.1)***^†^	92.5 (8.2)***^†††^	93.4 (10.3)***^†††^	92.7 (9.5)***^†††^
	≥20/day		90.6 (6.0)***	93.2 (7.9)***^†††^	94.2 (7.7)***^†††^	94.2 (7.7)***^†††^
	Current Smoker		89.3 (6.3)***	90.7 (8.0)***	90.7 (7.8)***	90.8 (8.1)***
	<20/day		88.8 (6.3)***	89.9 (7.5)***	90.0 (7.5)***	90.4 (8.0)***
	≥20/day		89.5 (6.2)***	91.0 (8.2)***	91.0 (7.9)***	91.0 (8.2)***

HbA1C (%)	Never Smoker		4.89 (0.3)^†††^	4.93 (0.3)^†††^	4.95 (0.3)^†††^	4.95 (0.3)^†††^
	Quitter		4.98 (0.4)	5.02 (0.4)*	5.03 (0.4)*	5.00 (0.4)^††^
	<20/day		4.94 (0.3)^†^	4.97 (0.3)	4.99 (0.5)^††^	4.99 (0.4)^†††^
	≥20/day		5.01 (0.4)	5.08 (0.4)	5.06 (0.3)^†^	5.02 (0.3)^††^
	Current Smoker		4.89 (0.4)***	4.91 (0.4)***	4.92 (0.4)***	4.92 (0.4)***
	<20/day		4.85 (0.3)***	4.87 (0.3)***	4.91 (0.3)***	4.91 (0.3)***
	≥20/day		4.92 (0.4)***	4.92 (0.4)***	4.93 (0.4)***	4.92 (0.4)***

Alcohol Consumption	Never Smoker		115.9 (123.3)^†††^	119.0 (127.4)^††^	114.4 (122.8)^††^	114.3 (123.6)^†††^
(mg/week)	Quitter		186.3 (158.7)	191.8 (196.3)	187.1 (164.4)^†^	188.7 (167.1)
	<20/day		156.1 (136.4)	162.3 (205.5)	159.5 (142.1)	158.9 (140.1)
	≥20/day		215.8 (173.4)	218.6 (182.0)	213.9 (178.5)	221.9 (185.5)
	Current Smoker		209.5 (177.3)*	214.5 (176.9)**	212.8 (173.1)***	212.7 (173.1)***
	<20/day		163.7 (146.3)**	170.1 (148.8)**	167.6 (144.5)***	176.9 (152.1)***
	≥20/day		226.3 (184.5)***	232.4 (183.7)***	233.2 (180.7)***	231.7 (180.4)***

Alcohol Drinking Rate	Never Smoker		58.7^&&&^	57.1^&&&^	55.3^&&&^	54.3^&&&^
(%)	Quitter		78.5^###^	74.9^###$^	74.9^###$^	74.5^###^
	Current Smoker		80.5^###^	79.9^###^	78.3^###^	77.4^###^

BMI, Body Mass Index; FPG, Fasting Plasma Glucose; HbA1c, Hemoglobin A1c.

Data are mean ± standard deviation (SD).

Significance was evaluated by 1-way ANCOVA with sex and age as covariates; post-hoc test (Bonferroni)

*P < 0.05 **P < 0.01 ***P < 0.001 (vs. never smokers), ^†^*P* < 0.05 ^††^*P* < 0.01 ^†††^*P* < 0.001 (vs. current smokers)

Significance was evaluated by chi-square test: ^#^*P* < 0.05 ^##^*P* < 0.01 ^###^*P* < 0.001 (vs. never smokers), ^$^*P* < 0.05 ^$$^*P* < 0.01 ^$$$^*P* < 0.001 (vs. never smokers)

## RESULTS

### Changes in BMI and rate of BMI increase during follow-up by smoking status

There was no significant difference in mean BMI at year 1 with regard to smoking status. From years 2 through 4, mean BMI was significantly higher in never smokers than in those with a history of smoking, but no significant difference was found between quitters and current smokers (Table 1). At year 2, the rate of BMI increase was significantly higher in quitters (2.36%) than in other groups (Table 2). At year 3, differences between quitters (<20/day) and the other groups became insignificant, but the rate was still significantly higher in quitters (≥20/day; 0.83%) than in other groups. At year 4, the rate in quitters did not significantly differ from those of other groups.

**Table 2.** Rate of BMI increase and number of subjects who developed Impaired Fasting Glucose, Impaired Glucose Tolerance, and Diabetes by year of examination and smoking status

	No. of cigarettes/day	No.	2nd year	3rd year	4th year
Rate of BMI increase% (SD)	Never Smoker		0.22 (3.2)	0.14 (3.2)^†^	0.12 (3.2)^†^
	Quitter		2.36 (3.9)***^†††^	0.46 (3.4)**	0.15 (3.1)
	<20/day		2.09 (3.8)***^†††^	0.20 (3.2)	0.00 (3.0)
	≥20/day		2.60 (4.1)***^†††^	0.83 (3.7)***^††^	0.30 (3.1)
	Current Smoker		0.29 (3.1)	0.32 (3.1)*	0.26 (3.1)*
	<20/day		0.32 (3.2)	0.48 (3.3)*	0.29 (3.0)
	≥20/day		0.27 (3.1)	0.25 (3.0)	0.25 (3.2)

		(Total)			
Impaired Fasting Glucose	Never Smoker	(9370)	935	906	913
(FPG ≥100 mg/dl) Incidence	Quitter	(946)	182	209	193
	<20/day	(472)	83	95	87
	≥20/day	(474)	99	114	106
	Current Smoker	(4976)	562	545	629
	<20/day	(1368)	120	128	155
	≥20/day	(3608)	442	417	474

Impaired Glucose Tolerance	Never Smoker		64	83	101
(FPG ≥110 mg/dl) Incidence	Quitter		18	29	29
	<20/day		10	14	14
	≥20/day		8	15	15
	Current Smoker		66	77	75
	<20/day		11	13	20
	≥20/day		55	64	55

Diabetes (FPG ≥126 mg/dl)	Never Smoker		1	8	10
Incidence	Quitter		0	2	3
	<20/day		0	1	3
	≥20/day		0	1	0
	Current Smoker		8	12	12
	<20/day		1	2	3
	≥20/day		7	10	9

BMI, Body Mass Index; FPG, Fasting Plasma Glucose.

Significance was evaluated by 1-way ANCOVA with sex and age as covariates, post-hoc test (Bonferroni)

*P < 0.05 **P < 0.01 ***P < 0.001 (vs. never smokers), ^†^*P* < 0.05 ^††^*P* < 0.01 ^†††^*P* < 0.001 (vs. current smokers)

### Characteristics of study subjects by smoking status

Mean FPG was significantly higher in quitters than in the other groups throughout the follow-up period. At year 1, HbA1c was not significantly different between quitters and never smokers, although it was significantly higher in quitters (<20/day) than in current smokers. At years 2 and 3, HbA1c in quitters was significantly higher than in never smokers, but the difference became insignificant at year 4. In comparison with never smokers, HbA1c in quitters was not significantly different at year 2; however, it was significantly higher at year 3 and thereafter. The amount and rate of alcohol consumption were significantly higher in quitters and current smokers than in never smokers in all years (Table 1).

### Odds ratios for incidence of IFG by smoking status

Before adjustment, the OR of IFG development among quitters was significantly lower, and that of current smokers was significantly higher, throughout the follow-up period. With regard to number of cigarettes per day, the OR was significantly lower in quitters (<20/day) but significantly higher in quitters (≥20/day) throughout the follow-up period. In contrast, the OR of current smokers (<20/day) was significantly higher throughout the follow-up period, and that of current smokers (≥20/day) was significantly lower in years 2 and 3. After adjustment for sex, age, BMI, FPG at year 1, and alcohol consumption, the OR of quitters was not significantly higher, while that of current smokers was significantly higher, throughout the follow-up period. With regard to number of cigarettes per day, the OR of quitters (<20/day) was not significantly higher throughout the follow-up period. In quitters (≥20/day), the OR was significantly higher at year 2 (OR 1.51) and year 3 (OR 1.71). The risk of IFG was not significantly increased at year 4 of follow-up in quitters regardless of the number of cigarettes. The OR of current smokers (<20/day) was significantly higher throughout the follow-up period, whereas the risk of IFG among current smokers (≥20/day) was not significantly increased throughout the period (Table 3). The OR of IGT after adjustment was 0.6 (95% confidence interval [CI], 0.4–0.95) in quitters and 0.57 (0.4–0.91) in quitters (<20/day) at year 2, which were significant decreases; however, no significant change was noted in other groups.

**Table 3.** Odds ratios for incidence of impaired fasting glucose and impaired glucose tolerance, by smoking status

Odds ratios for incidence of impaired fasting glucose (FPG ≥100 mg/dl)						

	2nd year		3rd year		4th year	
Odds Ratio (unadjusted)						
Never Smoker	Reference		Reference		Reference	
Quitter	0.75***	(0.7–0.85)	0.88*	(0.78–0.98)	0.74***	(0.66–0.83)
<20/day	0.67***	(0.6–0.76)	0.82***	(0.72–0.93)	0.71***	(0.62–0.8)
≥20/day	1.54**	(1.2–2.01)	1.92***	(1.48–2.48)	1.48**	(1.14–1.91)
Current Smoker	1.83***	(1.5–2.22)	2.32***	(1.93–2.8)	1.75***	(1.45–2.11)
<20/day	1.87***	(1.4–2.41)	2.43***	(1.9–3.11)	1.89***	(1.48–2.42)
≥20/day	0.68***	(0.5–0.84)	0.8*	(0.65–0.98)	0.83	(0.69–1)

Odds Ratio (adjusted)						
Never Smoker	Reference		Reference		Reference	
Quitter	0.81**	(0.7–0.95)	1.05	(0.89–1.25)	0.88	(0.75–1.04)
<20/day	0.75**	(0.6–0.89)	1.07	(0.89–1.29)	0.85	(0.71–1.02)
≥20/day	1.51*	(1.1–2.01)	1.71**	(1.23–2.38)	1.21	(0.87–1.68)
Current Smoker	1.64***	(1.3–2.08)	1.98***	(1.56–2.5)	1.36*	(1.08–1.72)
<20/day	1.57**	(1.1–2.14)	2.29***	(1.68–3.13)	1.38*	(1.02–1.87)
≥20/day	0.80	(0.6–1.03)	1.06	(0.82–1.37)	0.91	(0.73–1.15)


Odds ratios for incidence of impaired glucose tolerance (FPG ≥110 mg/dl)						

	2nd year		3rd year		4th year	

Odds Ratio (unadjusted)						
Never Smoker	Reference		Reference		Reference	
Quitter	0.51***	(0.4–0.73)	0.57**	(0.41–0.79)	0.721*	(0.53–0.98)
<20/day	0.45***	(0.3–0.65)	0.5***	(0.35–0.7)	0.699*	(0.5–0.98)
≥20/day	1.52	(0.8–3.01)	1.69	(0.92–3.11)	1.933*	(1.05–3.57)
Current Smoker	1.48	(0.9–2.54)	2.05**	(1.31–3.21)	2.182**	(1.4–3.4)
<20/day	1.22	(0.6–2.59)	1.86*	(1.03–3.36)	2.126*	(1.17–3.86)
≥20/day	0.52	(0.3–1.03)	0.54	(0.29–1.01)	0.942	(0.56–1.6)

Odds Ratio (adjusted)						
Never Smoker	Reference		Reference		Reference	
Quitter	0.60*	(0.4–0.95)	0.74	(0.48–1.14)	0.798	(0.52–1.23)
<20/day	0.57*	(0.4–0.91)	0.68	(0.43–1.09)	0.796	(0.5–1.27)
≥20/day	1.47	(0.7–3.21)	1.32	(0.63–2.79)	1.405	(0.66–2.98)
Current Smoker	1.35	(0.7–2.45)	1.56	(0.92–2.64)	1.456	(0.85–2.5)
<20/day	1.27	(0.6–2.76)	1.37	(0.69–2.72)	1.293	(0.63–2.67)
≥20/day	0.69	(0.3–1.44)	0.8	(0.41–1.57)	1.074	(0.58–1.98)

Data are shown as odds ratios (95% CI).

Signficance was evaluated by logistic regression (vs. Never Smoker) *P < 0.05 **P < 0.01 ***P < 0.001

Adjusted for Sex, Fasting plasma glucose at first year, Age, BMI, Alcohol Consumption

**Figure 2.**
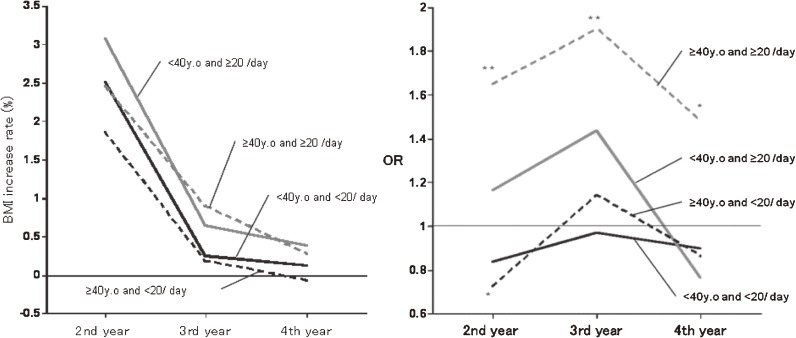
Time course of rate of BMI increase and odds ratio of IFG risk after smoking cessation. Significance was evaluated by logistic regression (vs. never smokers) *P < 0.05 **P < 0.01 ***P < 0.001 adjusted for sex, age, BMI, fasting glucose at first year, and alcohol consumption.

The subjects were divided into younger adults (39 years or younger) and middle-aged/elderly adults (40 years or older), and the rate of BMI increase and IFG risk after smoking cessation were investigated. The results are shown in Figure 2. The rate of BMI increase was highest after 1 year of smoking cessation (at year 2) regardless of the age or number of cigarettes, followed by year 3. The rate was lowest (<0.5%) in both groups at year 4. The OR for IFG risk was significantly higher throughout the period in the subjects aged 40 years or older who previously smoked 20 or more cigarettes a day (≥40 years and ≥20 cigarettes group; ORs: second year 1.7, third year 2.0, fourth year 1.5; Figure 2).

## DISCUSSION

No significant difference was noted in mean BMI with regard to smoking status at year 1. However, it was highest in quitters, lowest in never smokers, and moderate in current smokers at year 4 (Table 1). Previous studies have shown that quitters weighed more than current smokers and nonsmokers.^12^^,^^21^

After a review of 15 epidemiologic studies, a 1990 report of the US Surgeon General concluded that, during a median follow-up period of 2 years, average weight gain was 2.1 kg among quitters and 0.3 kg among continuing smokers and that smoking cessation resulted in 1.8-kg greater increase as compared with smokers.^21^ In our survey, BMI increased by 0.71 kg/m^2^ during 3 years of smoking cessation. This corresponds to a 2.05-kg increase, which is similar to the findings mentioned above (Table 1).

Weight gain occurs mainly during the first 2 years after quitting, and the effect is usually temporary.^3^ It has been reported that heavy smokers can experience considerable weight gain and that they weigh more than never smokers during the first few years after smoking cessation; however, they lose weight thereafter and return to the level of never smokers. Body weight in light and moderate smokers approaches that of never smokers, without any excess after smoking cessation.^3^ In contrast, in the present study, BMI in quitters (≥20/day) did not significantly differ from BMI in the other groups throughout the follow-up period, although the rate of BMI increase was significantly higher for 2 years after smoking cessation (at year 3). The rate of BMI increase in quitters (<20/day) was significantly higher for only 1 year after smoking cessation (year 2), but BMI was still significantly higher than that of never smokers after 3 years of smoking cessation (year 4; Table 1).

Increased food intake after cessation of smoking might be an important factor in weight gain. Weight gain after smoking cessation is explained by increased energy intake, decreased resting metabolic rate, decreased physical activity, and increased lipoprotein lipase activity.^22^^–^^24^ Withdrawal of nicotine can lead to increased appetite and excess caloric intake.^8^ In our survey, the difference in BMI between quitters and current smokers after 3 years of smoking cessation (year 4) was +0.46, which was not significant (Table 1). In another report, body weight and BMI decreased after long-term smoking cessation.^20^

In previous studies, mean FPG was significantly higher in quitters and current smokers than in never smokers throughout follow-up, but the risk of IFG after smoking cessation was not significantly higher than that in never smokers (Tables 1 and 3). In contrast to these previous reports,^15^^–^^17^ IFG risk did not increase after smoking cessation in the present study (Table 3). However, in another report, quitters did not have a significantly higher risk of diabetes as compared with never smokers,^9^ which is in conformity with our findings.

In current smokers, the OR for the risk of IFG at year 4 was 1.36 (95% CI, 1.08 to 1.72), which is significantly higher (Table 3). A recent meta-analysis^11^ of 25 prospective cohort studies with a follow-up ranging from 5 to 30 years reported a pooled adjusted relative risk for diabetes of 1.44 (95% CI, 1.31 to 1.58) for current smokers as compared with nonsmokers. Although our goals and follow-up period differed from those of previous studies, our finding of a 40% increase in the risk ratio of smokers is similar to their findings. In many previous reports, the risks of developing IGT and DM were elevated in smokers,^5^^–^^7^ as in our study. Smoking may lead to impaired endothelial function,^25^ which might result in reduced insulin sensitivity. In addition, oxidative stress is higher in smokers,^26^ and experimental evidence suggests that increased oxidative stress impairs insulin activity.^27^ The link between cigarette smoking and abnormalities in glucose homeostasis is biologically plausible, as several studies have suggested that smoking directly and indirectly impairs insulin sensitivity.^11^^,^^12^

The risk of IFG in quitters (≥20/day) was significantly increased after 1 and 2 years of smoking cessation, but the difference became nonsignificant after 3 years of cessation. These changes were consistent with those for the rate of BMI increase, which suggests that the risk of IFG in quitters (≥20/day) is associated with rate of BMI increase.

IFG risk in current smokers (<20/day) was higher than that in current smokers (≥20/day) throughout follow-up (Table 3). This is inconsistent with findings from previous reports, which showed both a significantly higher risk of diabetes among current smokers, as compared with never smokers, and a dose-response relationship between increases in smoking and the risk of diabetes.^28^^,^^29^ However, although Wannamethee et al^15^ also found that cigarette smoking was associated with an increased risk of diabetes after adjustment for confounders, they did not observe a dose-response relationship (adjusted relative risk: light smokers 1.79, moderate/heavy smokers 1.71). According to our findings, the risk of IFG in current smokers (<20/day) was 1.38 to 2.29, which was considerably higher than the risk of 0.8 to 1.06 in current smokers (≥20/day), which does not agree with the results of Wannamethee et al. This might be related to the rate of BMI increase, which was higher in current smokers (<20/day) (0.29–0.48) than in current smokers (≥20/day) (0.25–0.27), and to the anti-inflammatory effects of nicotine and its metabolites via the α-7 nicotinic acetylcholine receptors. The anti-inflammatory effects of white blood cells and adipocytes have been demonstrated in vitro^30^ and in vivo.^31^ Tjalve et al^32^ reported that nicotine inhibited glucose-induced insulin secretion at a high concentration and promoted insulin secretion at a low concentration. Reportedly, nicotine stimulates and inhibits parasympathetic ganglions at low and high concentrations, respectively, suggesting that nicotine influences insulin secretion via parasympathetic nerves.

After adjustment, the ORs for IGT were significantly reduced, at 0.6 (95% CI, 0.4–0.95) in quitters and 0.57 (0.4–0.91) in quitters (<20/day); however, no significant change was noted in the ORs for other groups. This is likely due to the rare occurrence of IGT at year 1, when subjects were limited to those with an FPG less than 100 mg/dL.

In an analysis of quitters subdivided into young (<40 years) and middle-aged/elderly (≥40 years) adults, the rate of BMI increase was highest after 1 year of smoking cessation (year 2) regardless of age or number of cigarettes, followed by that at year 3. The rate was lowest (<0.5%) in both groups after 3 years (year 4; Figure 2). Changes in the rate of BMI increase after smoking cessation were similar regardless of age or number of cigarettes. The ORs for IFG risk were significantly higher throughout the period in subjects aged 40 years or older who previously smoked 20 or more cigarettes a day (≥40 years and ≥20 cigarettes group; ORs: second year 1.7, third year 1.9, fourth year 1.5; Figure 2). Although BMI was highest after 1 year of smoking cessation in all groups, the ORs were highest after 2 years of smoking cessation (year 3). Changes in the risk of IFG were associated with changes in the rate of BMI increase, but lagged. IFG risk might thus be related to both the rate of change in BMI and its absolute value. The OR of older heavy smokers (≥40 years and ≥20 cigarettes group) after 3 years of smoking cessation was significantly higher, although it was lower than the OR after 2 years. However, because it was linked to changes in the rate of BMI increase, it is possible that it further decreased at year 4 of smoking cessation.

There were several limitations in this study. First, smoking habit was determined by using self-reports, as was the case in many similar studies. It has been reported that DM risk increases with exposure to secondhand smoke^33^; thus, it is desirable to objectively determine smoking status by using ELISA to measure urinary nicotine, a nicotine metabolite.^34^ Moreover, only subjects who underwent consecutive examinations were observed, and data concerning smoking were missing or incomplete in 4115 subjects (13.6% of 30 285), which might have resulted in bias. Furthermore, other lifestyle factors that might be associated with the risk of IFG, such as exercise amount and frequency, were not available, which also decreased the explanatory potential of this study. The maximum OR was 2.06 (Table 2). Zhang and Yu^35^ warn against implicit reliance on logistic regression ORs when the incidence of an outcome is higher than 10% and the OR is greater than 2.5. The number of quitters in the sample was 946. This condition could have been met if the sample size had been larger.

### Conclusion

The rate of BMI increase was significantly higher in quitters (2.36%) than in other groups (never smokers 0.22%, current smokers 0.29%) after 1 year of smoking cessation (year 2). After 2 years of smoking cessation (year 3), the rate of BMI increase in quitters (≥20/day) (0.83%) was significantly higher. The OR after adjustment for sex, age, BMI, FPG at year 1, and alcohol consumption was not significantly higher throughout the follow-up period. The risk of IFG among quitters (≥20/day) was significantly higher after 1 year (OR 1.51) and 2 years (OR 1.71) of smoking cessation. No significantly increased overall risk of IFG was observed after 3 years of smoking cessation in quitters regardless of the number of cigarettes per day previously smoked. However, when subjects were limited to those aged 40 years or older who previously smoked 20 or more cigarettes per day, the OR was significantly higher (1.5) at year 4. The risk of IFG after smoking cessation changed over time, similar to the rate of BMI increase. To maximize health improvements due to smoking cessation, we should advise all smokers to quit and offer smoking cessation treatment for those who want to do so. In addition, increases in blood glucose level and the risk of diabetes associated with smoking cessation-induced temporary weight gain should be monitored.
